# Fecal CD200 as a Measure of Immunosuppressive CD200L and Proinflammatory CD200S at the Feto‐Maternal Interface

**DOI:** 10.1111/aji.70120

**Published:** 2025-06-26

**Authors:** David A. Clark, Paul Moayyedi

**Affiliations:** ^1^ Department of Medicine Division of Clinical Immunology Faculty of Health, Sciences HSC 3H2 McMaster University Hamilton Ontario Canada; ^2^ Department of Pathology and Molecular Medicine Faculty of Health Sciences McMaster University Hamilton Ontario Canada; ^3^ Division of Gastroenterology Faculty of Health Sciences McMaster University Hamilton Ontario Canada

**Keywords:** CD200, cell degranulation, IBS‐D, irritable bowel syndrome, NK cells colon

## Abstract

**Problem:**

Patients with diarrhea‐predominant irritable bowel syndrome have an increased risk of recurrent miscarriage (RM). Loss of chromosomally normal post‐implantation embryos is triggered by the proinflammatory cytokines interferon‐γ and TNF‐α from NK cells and macrophages. Mouse models of RM similar to human unexplained RM have implicated fecal LPS as an important abortifacient. However, subnormal expression of immunosuppressive CD200L at the feto‐maternal interface has also been implicated in RM. Human stool contains desquamated epithelium expressing immunosuppressive CD200L and stromal CD56^+^ NK cells releasing proinflammatory CD200S^+^ granules. We asked if subnormal epithelial CD200L was associated with increased degranulation of stromal CD200^+^CD56^+^NK cells. Systemic effects would include an augmented proinflammatory milieu at the feto‐maternal decidual interface.

**Methods of Study:**

Quantitative analysis of biopsies of proximal and distal colon for immunostained for CD200L, CD200S, and CD56‐positive cells. CD200 ELISA Assay of stool extracts was done.

**Results:**

Epithelium and underlying stroma showed CD200L^+^ cells, CD200S^+^ cells, and CD56^+^ cells releasing CD200S‐positive granules. As epithelial CD200L expression decreased, the proportion of the degranulating CD56^+^ cells significantly increased. Degranulation was significantly greater in irritable bowel syndrome, diarrhea predominant subtype (IBS‐D) cases compared to controls. CD200L was detected in stool extracts.

**Conclusions:**

Decreased epithelial CD200L increased both CD66^+^ CD200S^+^ stromal cells and their degranulation. This implies potential functional effects. Stool CD200 may reflect the level of CD200 at the feto‐maternal decidual interface.

**Abbreviations**
IBS‐D: irritable bowel syndrome, diarrhea predominant subtype
LPS: lipopolysaccharide

## 1 Introduction

Investigation of the pathogenesis of recurrent miscarriages in mice has shown strikingly similar findings to data obtained from human recurrent miscarriage of chromosomally‐normal implanted embryos in humans [1]. In mice, fecal levels of endotoxin or LPS (lipopolysaccharide) have proven to be abortifacient, particularly when bowel permeability to LPS absorption is increased, as occurs in response to stress [1, 2]. Both in mice and humans, the level of the immunosuppressive form of CD200 (CD200L) at the feto‐maternal interface determines susceptibility to RM [1, 3]. There is also a pro‐inflammatory form of CD200 (CD200S) present in granules of endometrial CD56^+^ NK cells [4, 5].

A synergistic interaction between LPS‐sensitive macrophages and NK cells mediated by the cytokines TNF‐α an interferon‐γ augments the levels of these 2 cytokines, which are sufficient to activate blood coagulation, leading to ischemic death of implanted embryos in mice, and a similar process has been implicated in human RM [1, 6].

LPS activation of macrophages leads to RM by activating these processes, and CD200L serves to suppress this activation. It is logical to think that CD200S may have an opposite effect.

Irritable bowel syndrome has been linked to recurrent pregnancy loss and to subnormal levels of the immunosuppressive form of CD200 at the feto‐maternal decidual interface [7]. A substantial component of human feces is terminally differentiated colonic epithelial cells. Human colonic epithelial cells express CD200L [8]. We recently reported that decreased expression of the epithelial CD200L, the immunosuppressive form of CD200, led to increased frequency of CD56^+^stromal NK cells [7]. It is known in studies of uterine tissue that these CD56^+^ cells also express the pro‐stimulatory pro‐inflammatory molecule CD200S [5]. For these cells to exert their pro‐inflammatory function, it is hypothesized that the CD200S^+^ granules must be released and act on the CD200S receptor.

In this report, we show that the decreased epithelial CD200L expression in colonic epithelium is associated with increased degranulation of stromal (lamina propria) CD56^+^ CD200S^+^ NK‐type cells. CD200S from these cells is then able to exert proinflammatory effects on adjacent cells and on more distant targets. Local effects may include a leaky gut, which is associated with recurrent pregnancy loss [9].

## 2 Materials and Methods

The same cases described in the previous report were used and included non‐IBS controls, irritable bowel syndrome‐diarrhea predominant subtype (IBS‐D) cases, and microscopic colitis cases. Briefly, proximal and distal biopsies were done at the time of colonoscopy by Dr. Moayyedi. Four IBS‐D cases were male (M) age45, (M45), female age 58 (F58), F56, and M27. Controls were M55, F68, M21, and F69. Three microscopic colitis cases were included as a positive control. The slides were processed as previously described. Briefly, CD200L was detected using a 1/200 dilution of rabbit polyclonal antigen‐affinity‐purified IgG against amino acids (AA) 45–75 of he canonical P41217 (Q8TD46, Antibodies online ABIN761936). CD200S was detected at a 1/200 dilution of rabbit polyclonal antigen‐affinity‐purified IgG against AA ABIN318966. Although these AA are present in CD299L, using our staining methodology [10], these AA are only accessible when exon 2‐coded AA are deleted. The resultant molecule CD200S (S meaning shortened) has proinflammatory/pro‐rejection properties. After staining, a coverslip was applied to the slides were and the slides were scanned. The scans were analysed using ImageScope for CD200L and CD200S staining, and the proportion of stromal (lamina propria) cells that were granulated was calculated.

Calprotectin in stool extracts was tested for CD200 in an ELISA assay. Calprotectin is derived from neutrophils that invade the bowel wall in inflammatory bowel disease. Calprotectin was extracted using proprietary IDK extraction buffer diluted ½ in distilled water. The extraction buffer can also be made as 0.1 M Tris, 0.15 M NaCl, 1.0 M urea, 10 mM CaCl2, 0.1 M citric acid monohydrate, 5 g/L BSA, and 0.25 M thimerosal (pH 8.0). Stool samples were vortexed and after 10’ were centrifuged at 3000 × *g* for 10’ and supernatants were collected. The extracts were tested for calprotectin, and a value <70 is considered normal. Extracts were also tested for CD200 using a commercially available RaBioTech sandwich human ELISA kit (ELH‐CD200), RayBioTech, Norcross, GA, USA, as previously described. We do duplicate wells of undiluted and ½, and ¼ dilutions. With this ELISA, we have best results at a ½ sample dilution. The optical density was measured using BioTek Utility of Synergy H4 Hybrid Multi‐Mode Microplate Reader (BioTek, Winooski, VT) at a wavelength of 450 nm. CD200 concentrations were determined from a standard curve using different concentrations of the CD200 protein antigen standard (P41217.3), and the resulting optical density values were fitted to a second‐order polynomial *y* = ax^2^_+_ bx + c as described by Herman et al. using Microsoft Excel [11, 12]. Here *y* = OD, *x* = soluble CD200 concentration, and *c* = diluent alone. The concentration of CD200 unindividual test wells based on OD values was determined from the formula sCS200 pg/mL = (−b + SQRT[b^2^‐4a(c‐OD)])/(2a) With this method of analysis, using a standard curve from 6000 to 1.57 pg/mL, the reliable level of serum sCD200 was 12.5 pg/mL, although lower levels where OD values were > diluent negative control wells could be quantified with less precision.

As this was a retrospective tissue study without patient identifiers, informed consent was not required.

## 3 Results and Discussion

**FIGURE 1:**
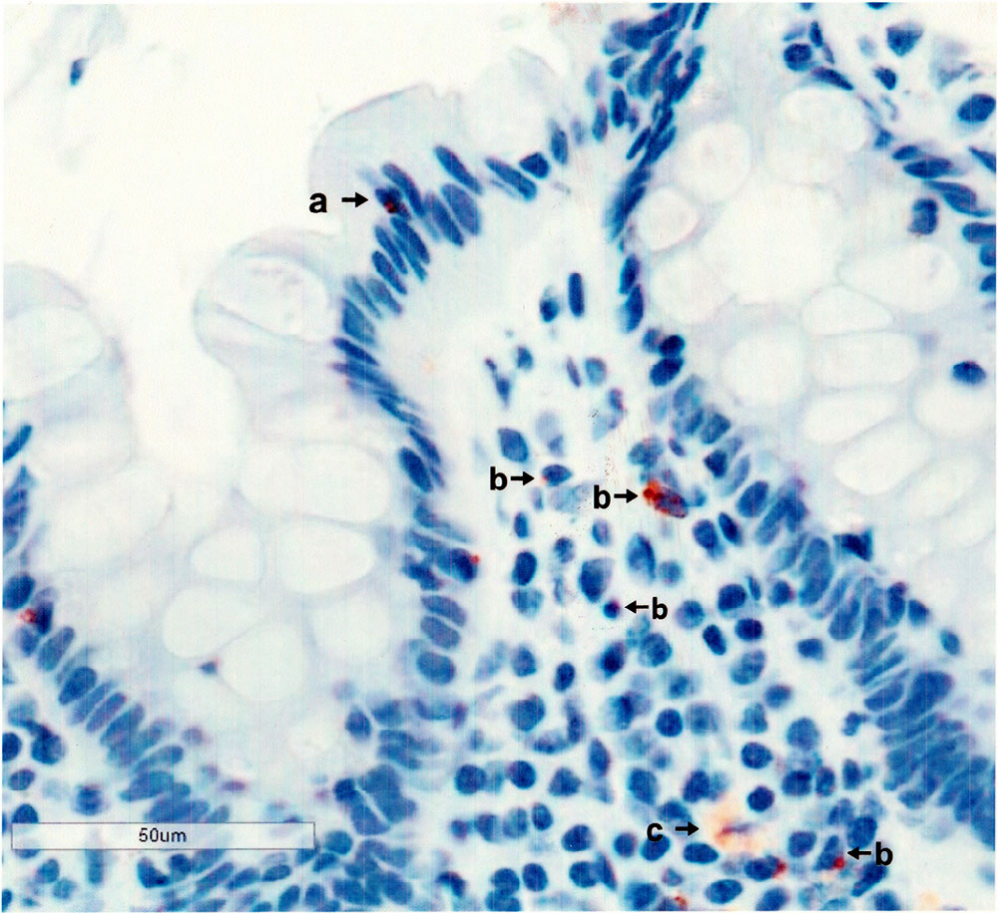
Biopsy of epithelium and underlying stroma stained as described in methods. Scale bar shows magnification. Typical control tissue.

As illustrated in Figure 1, cells staining for CD200S were detected in epithelium (arrow a). Similar cells were also detected in underlying stroma (arrow b). In some areas, as shown (arrow c), the cells had degranulated, and there was staining in the interstitium.

Figure 1 shows an example of epithelial and stromal standing. A scale bar is shown to indicate magnification.

**FIGURE 2:**
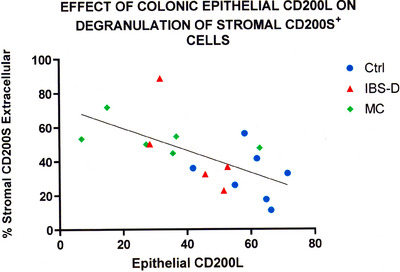
Correlation of CD200L epithelial staining intensity with underlying degranulation of CD56‐positive cells. Ctrl, non‐IBS control; IBS‐D, irritable bowel syndrome, diarrhea subtype; MC, microscopic colitis.

Figure 2 provides a correlation of the percent degranulation of stromal CD200S^+^ cells with epithelial CD200L expression intensity. Using individual implantation sites, we plotted the proportion of the degranulated CD200S^+^ cells with the level of expression of CD200L in epithelium. There was a negative correlation, and considering all 17 data points, the R value for the correlation coefficient of 0.47736 had a *p* value of less than 0.05%. One of the data points was previously shown to be an outlier due to excessively high expression of epithelial CD200L [1]. This was confirmed as an outlier using Grubb's test. The significance of the correlation in Figure 2 was not altered by eliminating that data point.

As we were primarily interested in whether used epithelial CD200L expression affected stromal degranulation in IBS‐D patients, we compared the percent degranulation in the control group to that in the IBS‐D group using Student's *t*‐test. The null hypothesis was that the degranulation in the IBS‐D group was not less than in the Control group. Including all five IBS‐D data values, there was significantly more degranulation in the IBS‐D patients with a *p* value of < 0.025. If the IBS‐D implantation site that had anomalously high epithelial expression of CD200L was omitted, the increased degranulation in the IBS‐D group was still statistically significant at *p* < 0.05.

A significant percentage of stool is composed of epithelial cells, and granules may also be present [3]. To determine if CD200 is present, an ELISA assay of calprotectin extracts was done. The results of sCD200 ELISA assay of 4 stool samples are shown in Table 1. Two of the 4 sample extracts contained statistically significant levels of CD200. One cannot determine using this ELISA if CD200L or CD200S was being detected. CD200S would represent granules released by CD56^+^ cells. It has been shown that percoll fractionation of stool can isolate viable colonic epithelial cells [3]. It should therefore be possible to measure colonic epithelial CD200L expression without the need for colonoscopy, biopsy, immunostaining, and a laborious microscopic quantitation procedure. That is important as variability in stool volume and consistency in any one individual could create considerable variability in the level of calprotectin and CD200, and it has not yet been shown that the calprotectin value can be used to adjust the value of CD200L.

**TABLE 1:** sCD200 levels in calpotectin supernatants.

Calprotectin level (mcg/gram of stool)	CD200 level (pg/mL)
21	0.0
10	20.1 ± 15.0
64	120.1 ± 20.0*
58	55.0 ± 10.0*

*Note:* * means significantly different from 0 with P < 0.05. compared to zero.

NK cells are present in human intestine, but there is limited information concerning their function and regulation [13]. Taken together with the results of our previous paper, our data suggests is associated with an increased frequency of stromal CD56^+^ CD200S^+^ NK cells and an increase proportion of which are discharging their granules into the interstitium.

CD200S^+^ cells are also present in the human endometrium [10]. Indeed, their number increases in the luteal phase when embryo implantation occurs. Given that there is a common mucosal immune system, and cellular events at one site cause similar changes at another site, it seems reasonable to suggest that colonic changes in CD200S‐positive cells will affect similar changes in the endometrium [14].

Taken together, one can appreciate that much can be learned about a patient from analysis of their stool. To understand the functional implications with respect to malfunction, it will be necessary to verify the ligands of CD200S and the physiological effects when it binds to receptors on adjacent cells. Distant effects seem possible given that endometrial CD200S‐positive cells degranulate with onset of menstruation, and in IBS‐D patients, diarrhea is more prominent at that time [10]. If colonic CD200S‐poitive cells affect the endometrium, one might expect to see more menorrhagia, and Jamieson and Steege reported among primary care practices of 533 patients, 90.4 % had menorrhagia (heavy bleeding) and 12% had irritable bowel syndrome (subtype not specified) [15]. Pati et al. had similar results [16]. So, additional data is worth collecting to determine if quantified menorrhagia is greater in women with IBS‐D. Finally, changes in CD200L and CD200S in the colonic epithelial cells in stool may inform about local pathology, such as IBS‐D and MC, and also about systemic changes, such as immunoregulation by CD200 at the feto‐maternal decidual interface that determines the fate of early pregnancy.

## Data Availability

The authors have nothing to report.
